# Neonatal sepsis at Muhimbili National Hospital, Dar es Salaam, Tanzania; aetiology, antimicrobial sensitivity pattern and clinical outcome

**DOI:** 10.1186/1471-2458-12-904

**Published:** 2012-10-24

**Authors:** Tumaini V Mhada, Francis Fredrick, Mecky I Matee, Augustine Massawe

**Affiliations:** 1 Department of Paediatrics, Bugando Medical Centre, P O Box 1370, Mwanza, Tanzania; 2 Department of Paediatrics and Child Health, School of Medicine, Muhimbili University of Health and Allied Sciences, P O Box 65001, Dar es Salaam, Tanzania; 3 Department of Microbiology and Immunology, School of Medicine, Muhimbili University of Health and Allied Sciences, P O Box 65001, Dar es Salaam, Tanzania

## Abstract

**Background:**

Neonatal sepsis contributes significantly to morbidity and mortality among young infants. The aetiological agents as well as their susceptibility to antimicrobial agents are dynamic. This study determined aetiology, antimicrobial susceptibility and clinical outcome of neonatal sepsis at Muhimbili National Hospital.

**Methods:**

Three hundred and thirty neonates admitted at the Muhimbili National Hospital neonatal ward between October, 2009 and January, 2010 were recruited. Standardized questionnaires were used to obtain demographic and clinical information. Blood and pus samples were cultured on MacConkey, blood and chocolate agars and bacteria were identified based on characteristic morphology, gram stain appearance and standard commercially prepared biochemical tests. Antimicrobial sensitivity testing was performed for ampicillin, cloxacillin, gentamicin, amikacin, cefuroxime and ceftriaxone on Mueller Hinton agar using the Kirby Bauer diffusion method.

**Results:**

Culture proven sepsis was noted in 24% (74/330) of the study participants. Isolated bacterial pathogens were predominantly *Staphylococcus aureus*, *Klebsiella spp* and *Escherichia coli. Klebsiella spp* 32.7% (17/52) was the predominant blood culture isolate in neonates aged below seven days while *Staphylococcus aureus* 54.5% (12/22) was commonest among those aged above seven days. *Staphylococcus aureus* was the predominant pus swabs isolate for both neonates aged 0–6 days 42.2% (98/232) and 7–28 days 52.3% (34/65). Resistance of blood culture isolates was high to ampicillin 81.1% (60/74) and cloxacillin 78.4% (58/74), moderate to ceftriaxone 14.9% (11/74) and cefuroxime 18.9% (14/74), and low to amikacin 1.3% (1/74). Isolates from swabs had high resistance to ampicillin 89.9% (267/297) and cloxacillin 85.2 (253/297), moderate resistance to ceftriaxone 38.0% (113/297) and cefuroxime 36.0% (107/297), and low resistance to amikacin 4.7% (14/297). Sepsis was higher in neonates with fever and hypothermia (p=0.02), skin pustules (p<0.001), umbilical pus discharge and abdominal wall hyperemia (p=0.04). Presence of skin pustules was an independent predictor of sepsis OR 0.26, 95% CI (0.10-0.66) p=0.004. The overall death rate was 13.9% (46/330), being higher in neonates with sepsis 24.3% (18/74) than those without 10.9% (28/256), p=0.003.

**Conclusions:**

*Staphylococcus aureus* was predominant isolate followed by *Klebsiella* and *Escherichia coli*. There was high resistance to ampicillin and cloxacillin. Mortality rate due to neonatal sepsis was high in our setting. Routine antimicrobial surveillance should guide the choice of antibiotics for empirical treatment of neonatal sepsis.

## Background

Neonatal mortality contribute significantly to the infant mortality rates in developing countries, various conditions are responsible for neonatal mortality among which is neonatal sepsis, which account for about 26% of neonatal mortality [1]. About four million neonatal deaths occur worldwide with reported highest rates in Sub-Sahara Africa [1].

Neonatal sepsis which is defined as sepsis occurring in the first 28 days of life can be divided into early onset and late onset. Differentiation into early and late onset neonatal sepsis is important in prevention and treatment because of aetiological differences. Very few studies in sub-Sahara Africa has looked into neonatal sepsis with the focus on time of onset [2,3]. In this study we defined early onset neonatal bacterial sepsis as sepsis in neonates less than seven days and late onset as sepsis in 7 days or more.

Clinical presentation of neonatal sepsis varies and there are no pathognomonic features [4], however some clinical features have been reported to predict sepsis. Kayange et al. in a study which was conducted in Bugando, Tanzania reported inability to breast feed, lethargy, convulsion, chest wall in-drawing, jaundice and umbilical redness to be strongly associated with neonatal sepsis [5]. Non-specific presentation of neonatal sepsis and poor or delayed laboratory services have resulted in empirical treatment of sepsis in resource limited set up. In a study conducted in Kilimanjaro, Tanzania more than two thirds of neonates admitted to a special care baby unity received antibiotics [6].

In the management of neonatal sepsis, clinicians in many resource limited settings make tentative diagnosis and empirical treatment of neonatal sepsis based on the new neonatal WHO Integrated Management of Childhood Illnesses (n-IMCI) guidelines [7]. However aetiology of neonatal sepsis as well as response to antimicrobial agents may vary significantly from time to time and geographically which may affect the success of empirical management [5,7-10].

Currently at Muhimbili National Hospital, the largest referral hospital in Tanzania, neonatal sepsis is managed according to WHO guideline which recommends ampicillin, cloxacillin and gentamycin as the first line antibiotics [11]. Reports of dynamic nature of the aetiological agents and their response to antimicrobial agents in different geographical areas calls for availability of local data in guiding choices of antibiotics. This study was carried out to determine aetiology, antimicrobial resistance of isolated bacteria and outcome of neonatal sepsis.

## Methods

This was a hospital based cross-sectional study carried out at Muhimbili National Hospital neonatal unit. Both term and preterm neonates admitted with clinical diagnosis of neonatal septicaemia, presenting with any one of the following features were eligible; presence of fever (≥ 38.0°C) or hypothermia (≤ 36.5°C), convulsions, lethargy, inability to feed, hypoglycaemia, vomiting, bulging fontanels, respiratory distress, jaundice and signs of infection on the skin (pus spots) and umbilical pus discharge or hyperaemia. Sample size in this study was calculated using *Epi info version 6* statistical package, and a prevalence of 16% from a study conducted previously at the same hospital by Bloomberg et al. [10] was used with a target of recruiting about 300 participants. During the study period 1811 were admitted in the neonatal ward out of which 330 fulfilled inclusion criteria and were recruited.

Standardized questionnaires were used to obtain demographic and clinical information which included details of thorough physical examination. Weight was measured using SECA beam balance (CMS Weighing equipment, London, United Kingdom) to the nearest 100g. Two millilitres of venous blood was aseptically drawn from anterior cubital fossa of each neonate and inoculated into paediatric blood culture bottles. Pus swab were taken from the umbilical cord stump or skin pustules to determine the contribution of cord stump/skin infection to sepsis and correlate the two infections, pus swabs were transported in Stuart transport media.

Blood culture bottles were incubated at 37°C for 24 h after which aliquots were sub-cultured on solid agar plates; MacConkey, blood and chocolate agars for up 96 h before being regarded as no growth. Pus swabs were smeared on a glass slides for gram stain identification and were also inoculated on solid MacConkey agar at 37°C for 24 h. Colonies on solid agar plates were identified based on characteristic morphology, gram stain appearance and standard commercially prepared biochemical tests [12].

Antimicrobial sensitivity testing was performed for antimicrobials which included first and second line antibiotics for treating neonatal sepsis at MNH; first line antibiotics are ampicillin, cloxacillin and gentamicin and second line is ceftriaxone. Ampicillin 10μg, cloxacillin 5μg gentamicin 10μg, amikacin 30μg, cefuroxime 30μg, and ceftriaxone 30μg sensitivity testing were performed by Kirby Bauer diffusion method using Mueller Hinton agar with incubation of 24 h at 37°C. Sensitivity was determined according to Clinical Laboratory Standard Institute standards [13]. Results were recorded as resistant, intermediate and sensitive, however, during data analysis intermediate were categorized as resistant.

Statistical Package for Social Sciences (SPSS) version 17 was used for data entering, cleaning and analysis. Chi square and Fischer’s exact test were used to determine association between categorical variables. Probability (p) value of <0.05 was considered statistically significant. This study was approved by MUHAS ethical committee and informed written consents were obtained from parents/guardian prior to recruitment. Results of laboratory tests were used in the participants’ management.

## Results

### Demographic and clinical characteristics of participants

Three hundred and thirty neonates with a median age of 3 days (range 0–26) were recruited. Hundred and sixty (48.5%) were female and 253 (76.7%) were aged between zero and six days. Table 1 shows demographic characteristics of participants. Most frequently reported clinical features of included pus discharge with hyperaemia (96.1%) and fever (91.5%). Other included inability to breastfeed, lower chest wall in-drawing, difficulty in breathing, jaundice, bulging fontanelle, skin pustules, convulsion and hypothermia (Table 1).

**Table 1:** Demographic and clinical characteristics of participants

**Variables**	**N=330**	**%**
**Age (Days)**		
0 – 6	253	76.7
7 – 28	77	23.3
**Sex**		
Male	170	51.5
Female	160	48.5
**Weight (gm)**		
≤ 2500	109	33
> 2500	221	67
**Maturity**		
Preterm	77	23.3
Term	253	76.7
**Clinical features (signs & symptoms)**		
Fever	302	91.5
Hypothermia	8	2.4
Inability to feed	206	62.4
Bulging fontanelle	33	10.0
Difficulty in breathing	73	22.1
Lower chest wall in drawing	89	27.0
Convulsions	10	3.0
Umbilical pus discharge with hyperaemia	317	96.1
Skin rash with pus spots	31	9.4
Jaundice	44	13.3

### Bacterial isolation

Three hundred and two participants 91.5% (302/330) had culture proven bacterial infection out of which 69.1% (228/330) had bacteria isolated from swabs, 1.5% (5/330) had bacteria isolated from blood and 20.9% (69/330) had bacteria isolated from both swabs and blood. One hundred and ninety eight (66.7%) swabs were taken from cord stump while 33.3% (99/297) were taken from skin pustules. Among 69 participants who had bacteria isolated from both swabs and blood 65.2% (45/69) had the same bacteria isolated from both swabs and blood and 34.8% (24/69) had different organisms isolated. Therefore culture proven sepsis was present in 22.4% (74/330).

*Staphylococcus aureus* was the most common isolated organism from blood culture and pus swab, followed by *Klebsiella species* and *Escherichia coli.* These three organisms accounted for approximately 85% of all isolates in blood culture and 94.3% of all isolates from pus swab. For bacteria isolated from blood *Klebsiella spp* 32.7% (17/52) was predominant at the age 0 to 6 days where as *Staphylococcus aureus* 54.5% (12/22) featured more among neonates aged 7–28 days. (Table 2). *Staphylococcus aureus* was the predominant isolate from pus swabs in both 0–6 days 42.2% (98/323) and 7–28 days group 52.3% (34/65) (Table 3).

**Table 2:** Bacterial isolation from blood

**Organism**	**Early and late infection (days) (N=74)**		**Total N(%)**	**p value**
	**0 – 6 n(%)**	**7 – 28 n(%)**		
*Staphylococcus aureus*	15 (55.6)	12 (44.4)	27 (36.5)	0.721*
*Klebsiella species*	17 (77.3)	5 (22.7)	22 (29.7)	
*Escherichia coli*	10 (71.4)	4 (28.6)	14 (18.9)	
*Staphylococcus epidermidis*	6 (100)	0 (0)	6 (8.1)	
*Group B Streptococci*	1 (100)	0 (0)	1 (1.4)	
*Pseudomonas spp*	1 (50.0)	1 (50.0)	2 (2.7)	
Other *streptococcus species*	2 (100)	0 (0)	2 (2.7)	
**Total**	**52 (70.3)**	**22 (29.7)**	**74 (100)**	

*Fisher’s exact test.

**Table 3:** Bacterial isolation from pus swabs

**Organism**	**Early and late infection (days) (N=297)**		**Total n (%)**	**p value**
	**0 – 6 n (%)**	**7 – 28 n (%)**		
*Staphylococcus aureus*	98 (74.2)	34 (25.8)	132(44.4)	0.616*
*Klebsiella species*	61 (79.2)	16 (20.8)	77 (25.9)	
*Escherichia coli*	57 (80.3)	14 (19.7)	71 (23.9)	
*Staphylococcus epidermidis*	3 (100)	0 (0)	3 (1.0)	
*Pseudomonas species*	6 (85.7)	1 (14.3)	7 (2.4)	
*Proteus species*	7 (100)	0 (0)	7 (2.4)	
**Total**	**232(78.1)**	**65 (21.9)**	**297 (100)**	

*Fisher’s exact test.

### Antimicrobial susceptibility of isolated bacteria

Antimicrobial susceptibility pattern was performed for all bacterial isolates. Isolates from both blood and pus swabs had highest resistance against ampicillin and cloxacillin. All *Klebsiella spp* isolates and 92.9% (13/14) of *Escherichia coli* isolates from blood were resistant to ampicillin, 77.0% (12/22) of *Klebsiella spp* and 42.9% (6/14) of *Escherichia coli* isolated from blood were resistant to gentamycin. Twenty two (81.5%) of *Staphylococcus* aureus isolated from blood were resistant to cloxacillin. Table 4 shows antimicrobial resistance pattern of organisms isolated form blood culture.

**Table 4:** Antimicrobial resistance pattern of isolated bacteria from blood (N=74)

**Organism**	**Antibacterial agent**					
	**Amp R n (%)**	**Clox R n (%)**	**Gent R n (%)**	**Amik R n (%)**	**Cefur R n (%)**	**Ceft R n(%)**
*Staphy. aureus*	23 (85.2)	22 (81.5)	15 (55.6)	0 (0)	4 (14.8)	4 (14.8)
*Klebsiella species*	22 (100)	21 (95.5)	17 (77.3)	1 (4.5)	8 (36.4)	4 (18.2)
*E. coli*	13 (92.9)	14 (100)	6 (42.9)	0 (0)	1 (7.1)	2 (14.3)
*Group B Streptococci*	0 (0)	1 (100)	1 (100)	0 (0)	0 (0)	0 (0)
*Pseudomonas spp*	1 (50)	0 (0)	0 (0)	0 (0)	1 (50)	1 (50)
Other *Strept. species*	1 (50)	0 (0)	1 (50)	0 (0)	0 (0)	0 (0)
**Total**	**60 (88.2)**	**58 (85.3)**	**40 (58.8)**	**1 (1.5)**	**14 (20.6)**	**11 (16.2)**

Amp: Ampicillin, Clox: Cloxacillin, Gent: Gentamycin, Amik: Amikacin, Cefur: Cefuroxime, Ceft: Ceftriaxone, R: Resistance.

Of the isolated *Klebsiella spp* from swabs 97.4% (75/77) were resistant to ampicillin and 88.7% (63/71) of *Escherichia coli* isolates from swabs were resistant to ampicillin. Gentamycin resistance from *Klebsiella spp* and *Escherichia coli* were noted in 61% (47/77) and 39.4% (28/71) respectively. Of the *Staphylococcus aureus* isolates from swabs 80.3% (106/132) were resistant to cloxacillin (Table 5).

**Table 5:** Antimicrobial resistance pattern of isolated bacteria from swabs (N=2974)

**Organism**	**Antibacterial agent**					
	**Amp R n (%)**	**Clox R n (%)**	**Gent R n (%)**	**Amik R n (%)**	**Cefur R n (%)**	**Ceft R n (%)**
*Staphy aureus*	116 (87.9)	106 (80.3)	69 (52.3)	7 (5.3)	45 (34.1)	55 (41.7)
*Kleb species*	75 (97.4)	75 (97.4)	47 (61)	2 (2.6)	34 (44.2)	31 (40.3)
*E. coli*	63 (88.7)	60 (84.5)	28 (39.4)	5 (7)	24 (33.8)	19 (26.8)
*Pseudomonas spp*	7 (100)	7 (100)	4 (57.1)	0 (0)	4 (57.1)	6 (85.7)
*Proteus species*	6 (85.7)	5 (71.4)	2 (28.6)	0 (0)	0 (0)	2 (28.6)
**Total**	**267(90.8)**	**253 (86.1)**	**150 (51.0)**	**14 (4.8)**	**107(36.4)**	**113 (38.4)**

Amp: Ampicillin, Clox: Cloxacillin, Gent: Gentamycin, Amik: Amikacin, Cefur: Cefuroxime, Ceft: Ceftriaxone, R: Resistance.

### Clinical features

Participants with fever 21.9% (66/302) and hypothermia 62.5% (5/8) were noted to have higher frequency of sepsis as compared to those with normal body temperature 15.0% (3/20), p=0.02. Occurrence of sepsis was also noted to be higher among neonates who had skin rash with pustules 16/31 (51.6%) as compared to those without skin rashes 58/299 (19.4%),p<0.001 . Presence of skin rashes with pustule was an independent predicator of sepsis [OR 0.26, 95% CI (0.10-0.66), p=0.004].

### Immediate outcome of sepsis

Forty-six (13.9%) neonates recruited in this study died. Thirty six neonates out of 253 (14.2%) aged between 0–6 and 10/77 (13%) aged between 7–28 days died. Neonates with sepsis were noted to have significantly higher proportion of deaths 18/74 (24.3%) as compared to those without sepsis 28/256 (10.9%) p=0.003. No association was noted between type of isolated bacteria and death p=0.196 (Fisher’s exact test).

## Discussion

From the findings of this study, neonatal sepsis is common and contributes significantly to mortality among neonates admitted in the neonatal ward at Muhimbili National Hospital. The prevalence of positive blood culture sepsis of 22.4% observed in this study is higher than 15.9% reported by Bloomberg et al. [10] from a study conducted earlier at this hospital. It is also higher than 6.5% which was reported by Klingenberg et al. [6] from a study conducted in another Tanzanian referral hospital. The increase in prevalence of sepsis could be accounted for by the increase in resistance to antimicrobial which may have resulted in inadequate treatment in lower health care facilities. Collectively, our study and that of Bloomberg et al. [10] indicate a high magnitude of neonatal sepsis at MNH, which may reflect low quality of neonatal care as opposed to developed countries which have lower prevalence rates of neonatal sepsis [7,14].

### Clinical outcome of neonatal sepsis

In this study, the overall neonatal mortality was 13.9% (46/330), being higher in neonates with sepsis (24%) as compared to those without (10%). Reports from other two referral hospitals in Tanzania by Kayange et al. [5] and Klingenberg et al. [6] found infant mortality due to neonatal sepsis to be 19% and matched closely with the findings of 18% by Mugalu et al. in a Ugandan study [15]. Collectively, these findings reflect the significant contribution of neonatal sepsis in both neonatal and infant mortality.

### Aetiology of neonatal sepsis

We found *Staphylococcus aureus*, *Klebsiella spp* and *Escherichia coli* to be the predominant bacterial isolates, which is in keeping with findings from a number of studies conducted in sub-Saharan Africa [5,16-19]. *Klebsiella* spp was the main isolate in early onset sepsis while *Staphylococcus aureus* dominated in late onset sepsis and this is similar to report by Mokuolu et al. in a study which was conducted in Nigeria [17]. Organisms isolated with respect to time of onset in this study seem to fit with the proposed sources of bacteria in neonatal sepsis which are birth canal for early sepsis in which *Klebsiella spp* was the predominant isolate, and hospital sources or community for late sepsis in which *Staphylococcus aureus* was predominant isolate, respectively [7,19]. *Staphylococcus aureus* was the main isolate from swabs which may be attributed to acquisition of bacteria through handling of the neonates by health care providers and family members.

### Antimicrobial susceptibility of bacterial isolates

Our findings show that most of the bacterial isolates showed high resistance to ampicillin, cloxacillin and gentamycin which are first line antimicrobials for treating neonatal sepsis at our centre. More than 80% of *Staphylococcus aureus* and more than 90% of *Klebsiella* spp from both blood and swabs were resistant to ampicillin and cloxacillin, while more than 50% of *Staphylococcus aureus* and more than 60% of *Klebsiella* spp were resistant to gentamycin. High resistance noted may be attributed to excessive and irrational use of these antibiotics at primary health facilities from which neonates are referred to our centre.

Notably, the resistance of all isolates to ceftriaxone, cefuroxime and amikacin was significantly low. At MNH ceftriaxone is used a second line, while cefuroxime and amikacin are not included in the protocol of neonatal sepsis management. From our findings and other studies in the region showing high levels of antimicrobial resistance to ampicillin, cloxacillin and gentamycin [6,18,20] it is apparent that the current antimicrobial regiment for empirical treatment of neonatal sepsis need to be revisited. Amikacin and cefuroxime may be alternative choice of antimicrobials in empiric treatment of neonatal sepsis however these will be more expensive.

### Study limitations

Some of the study participants referred to our hospital might have been inadequately treated resulting in selection bias in our study. Anaerobes which may cause sepsis and other infections with similar presentation to sepsis including malaria [21] were not looked for in this study. *Staphylococcus epidermidis* isolated in early neonatal sepsis 6/52 (11.5%) may be due to contamination during blood collection [22,23]. Extended beta lactamase resistant strains were not determined and resistance to methicillin by *Staphylococcus aureus* isolates were not determined in this study.

## Conclusions

*Staphylococcus aureus* and *Klebsiella spp* were the predominant bacterial isolates in this study and showed high resistance to ampicillin and cloxacillin which are WHO recommended and routinely used antimicrobial at MNH. This calls for institutional based antimicrobial surveillance to guide choices of antibiotics for treating neonatal sepsis.

## Competing interests

Authors declare that they have no competing interests.

## Authors’ contributions

TVM designed the study, collected data, analyzed data and participated in manuscript preparation. FF assisted in data collection, analysis and prepared the manuscript. MIM and AM critically reviewed the study protocol, supervised data collection and participated in manuscript preparation. All authors have read and approved the final manuscript.

## Pre-publication history

The pre-publication history for this paper can be accessed here:

http://www.biomedcentral.com/1471-2458/12/904/prepub
